# Co-design of a digital violence prevention and management tool for psychiatric inpatient care: focus on supporting integration into electronic health record system

**DOI:** 10.1192/j.eurpsy.2024.170

**Published:** 2024-08-27

**Authors:** T. Lantta, T. Rautiainen, M. Anttila, J. Anttila, M. Ameel

**Affiliations:** ^1^Department of Nursing Science, University of Turku, Turku, Finland; ^2^Centre for Forensic Behavioural Sciences, Swinburne University of Technology, Melbourne, Australia; ^3^Hospital Distric of Helsinki and Uusimaa; ^4^University of Helsinki, Helsinki, Finland

## Abstract

**Introduction:**

Violence in psychiatric inpatient settings is a global challenge. Several methods have been developed and tested to help staff prevent the occurrence of violence on the wards. One novel and effective method is eDASA+APP, originating from Australian forensic psychiatric settings (Maguire *et al.* Int J Ment Health Nurs 2019; 281186-1197, Griffith *et al*. Psychiatr Serv 2021; 72 885-890). This electronic method contains an instrument (DASA) to assess the risk for imminent violence and includes evidence-based violence risk management methods for risk levels. It is important to ensure that this electronic intervention is integrated into daily clinical practice. This can be done in co-design between all that are involved e.g., staff and experts by experience, and by encouraging them to achieve a common goal and gain benefits by working together.

**Objectives:**

This prevention gives an overview of how the Finnish version of eDASA+APP was co-designed with healthcare staff and experts by experience, focusing on integration into the electronic patient health record system. The presentation is part of a larger research project testing eDASA+APP in Finnish psychiatric inpatient care.

**Methods:**

Co-design workshops focusing on three major themes: 1) identifying current practices and how eDASA+APP would fit in those, 2) producing a linguistically and culturally appropriate version of eDASA+APP, and 3) preferred use of eDASA+APP in an electronic patient health record system. Notes were kept during the workshops by researchers. Qualitative material were analysed with deductive content analysis. Results from the third theme are shared in this presentation.

**Results:**

Staff and experts by experience described that integration of eDASA+APP in electronic patient health record system is supported if it 1) brings clear and fast information to the staff about the violence risk of a patient, 2) is a visible measure that is concretely in sight in electronic patient health record system, 3) provides information about which violence prevention and management interventions have worked with a patient, 4) involves patient preferences, and 5) consist of joint decisions that have been agreed multi-professionally.

**Conclusions:**

Integration of eDASA+APP in the electronic patient health record system has the potential to succeed if it is realized in cooperation with staff and experts by experience, is technically easy to use, and the users have an understanding of its benefits to everyone involved.

**Disclosure of Interest:**

None Declared

